# Parameter Optimization Based BPNN of Atmosphere Continuous-Variable Quantum Key Distribution

**DOI:** 10.3390/e21090908

**Published:** 2019-09-18

**Authors:** Yu Su, Ying Guo, Duan Huang

**Affiliations:** 1 School of Computer Science and Engineering, Central South University, Changsha 410083, China; 2 School of Automation, Central South University, Changsha 410083, China

**Keywords:** continuous variable quantum key distribution, atmosphere turbulence, machine learning, back-propagation neural network

## Abstract

The goal of continuous variable quantum key distribution (CVQKD) is to be diffusely used and adopted in diverse scenarios, so the adhibition of atmospheric channel will play a crucial part in constituting global secure quantum communications. Atmospheric channel transmittance is affected by many factors and does not vary linearly, leading to great changes in signal-to-noise ratio. It is crucial to choose the appropriate modulation variance under different turbulence intensities to acquire the optimal secret key rate. In this paper, the four-state protocol, back-propagation neural network (BPNN) algorithm was discussed in the proposed scheme. We employ BPNN to CVQKD, which could adjust the modulation variance to an optimum value for ensuring the system security and making the system performance optimal. The numerical results show that the proposed scheme is equipped to improve the secret key rate efficiently.

## 1. Introduction

The flying start of quantum communication makes secure communication conceivable in practice [1,2,3]. As an important applicatory adhibition in the quantum communications, quantum key distribution (QKD) permits communication objects to generate a public secret key at the existence of eavesdropping, and this approach implements secure key interchange that does not rely on computational complexity [4,5,6,7,8,9]. Discrete-variable quantum key distribution (DVQKD) as well as continuous-variable quantum key distribution (CVQKD) are two primary ways to implement QKD. DVQKD protocol demands greatly faint light pulses in the process of generation and detection [5,6]. Another method of protocol, CVQKD, can utilize standard components of fiber optic communication without the need for a single photon detector [7,8,9,10].

Nowadays, QKD has entered a new stage of development with the goal of widespread application and adoption under a variety of environmental conditions. Long distance QKD in the atmospheric turbulence channel has been realized [11,12,13]. Experimental advances in this field have enabled the transmission of quantum light over horizontal communication links to be shown to be successful [14,15,16,17,18,19], and the satellite-to-ground DVQKD has been confirmed to exceed 1200 km [20]. Schemes employing single-photon detectors are influenced by background noise [21]; at the same time, coherent detection uses a bright local oscillator (LO) operating as a filter to decrease the background noise [22].

In the process of atmospheric propagation, the beam as a whole experiences random broadening, deformation and random deflection. The main influence comes from the turbulence fluctuation of a refractive index. In addition, the beam can be weaken by backward scattering and absorption. For weak turbulence, the atmosphere mainly causes beam wandering, and this case may be well depicted by the log-negative Weibull distribution [23], while, for moderate and strong turbulence, the beam will be stretched and deformed to form a smooth probability distribution of the transmittance (PDT). In addition, the elliptic-beam model gives consistent results with this situation [24]. Furthermore, this model can also be employed to analyze atmospheric quantum channels under different weather circumstances [25].

The secure key rate depends on the modulation variance, transmittance and excess noise. Unlike optical fiber channels, atmospheric channel transmittance is affected by many factors and does not vary linearly. Therefore, it is crucial to select a suitable modulation variance for different turbulence intensities. This is a kind of a parameter optimization that could guarantee the system security and make the system performance optimal. Previously, to obtain the optimal parameters, it always recurred to simulation and iteration. With enough time and iteration in advance, the approach is mature and accurate [26,27]. Nevertheless, emulators often consume a large amount of time, notably when running optimizers, which must be repeated many times. Parameter optimization becomes more complex when we extend point-to-point communication to communication networks.

Machine learning provides formidable implements for settling matters in many fields, such as estimating parameters, output forecast on the basis of previous input data, data sorting, and pattern identification [28,29]. In recent years, machine learning technology has been diffusely used both in coherent optical communication [30,31,32,33,34] and QKD systems [35,36,37,38,39]. The support vector regression (SVR) is applied to predict the time-along intensity evolutions of the laser light and the LO pulse to improve system performance [35]. In addition, for parameter optimization, this has been done with machine learning in measurement-device-independent (MDI) DVQKD [38,39]. Hence, the above-mentioned methods provide an idea for parameter optimization in CVQKD at the atmospheric turbulence channel.

In this paper, we propose an approach that employs a back propagation artificial neural network (BPNN), which is one of the most popular machine learning tools. The theory of BPNN is described in detail, and parameter optimization on atmospheric channels is proposed. In parameter optimization, BPNN is mainly used to predict, rather than search, the optimized parameter. We employ BPNN to CVQKD, which could adjust the modulation variance to an optimal value to ensure the system security and make the system performance optimal. The numerical results show that the proposed scheme is equipped to improve the secret key rate efficiently.

The structure of the paper is listed next. In Section 2, we briefly introduce the transmission models under different intensities of turbulence and the security analysis—whereafter, the Monte Carlo method is employed to estimate transmittance distribution of the atmospheric turbulence channel. In Section 3, we propose the BPNN-based CVQKD scheme. Section 4 verifies the performance improvement and security analysis of the CVQKD system with numerical simulations. In the end, the conclusions are drawn in Section 5.

## 2. Transmittance and Security Analysis

In this section, the main purpose is to analyze the security of the atmospheric channel. Due to the fluctuating of the atmospheric channel, we first analyze the transmittance of the channel according to different turbulence intensity. In addition, we then apply the transmittance analyzed above to the calculation of the secret key rate, in order to obtain the CVQKD security analysis under the atmospheric channel.

The Rytov variance is used for depicting the turbulence intensity, and its expression is [40,41]

(1) $$\sigma_{R}^{2}=1.23C_{n}^{2}k^{7/6}L^{11/9},$$

where $C_{n}^{2}$ is the refraction index structure parameter, *k* denotes the optical wave number, and *L* represents the horizontal propagation distance. In the case of horizontal propagation, $C_{n}^{2}$ can be seen as a constant. As shown in Table 1, the value of $C_{n}^{2}$ we used is on the basis of long-term radiosonde measurements in Hefei, Anhui, China [42].

### 2.1. Transmittance Analysis

In a weak turbulent atmosphere, beam-wandering plays a primary role. Generally, they are induced by unstable adjustment of a radiation source and aperture truncation of the light at the receiving end [43]. The characterization of the beam wandering mechanism and channel models of fluctuation are demonstrated in [23].

**Figure 1:**
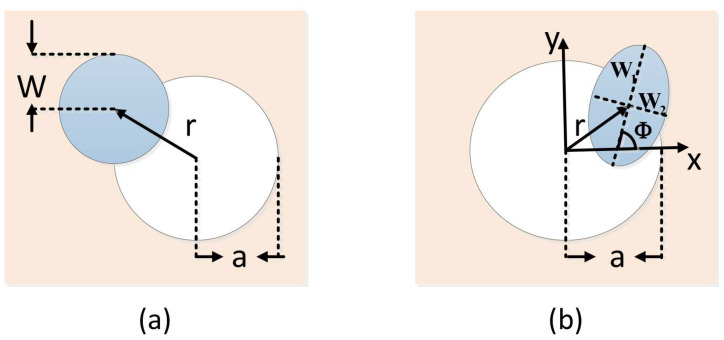
(**a**) in the beam wandering case, the fluctuation of transmittance results from the variation of the beam-deflection distance *r*; (**b**) the elliptical beam profile is characterized with the half axis $W_{1}$ and $W_{2}$, where $W_{1}$ rotates on the angle Φ relating to the *x*-axis. Beam wandering is characterized by parameter *r*, which represents the beam-centroid position with respect to the center of the aperture.

Under the circumstance of beam wandering (see Figure 1a), an approximate analytical representative of transmission efficiency can be expressed as

(2) $$T^{2}=T_{0}^{2}\exp\left[-{\left(\frac{r}{R}\right)}^{\lambda }\right],$$

where *r* denotes the distance of beam-deflection. $T_{0}$ denotes the transmission coefficient maximum for the specified beam-spot radius value *W*, *R* and λ are scale and shape parameters. The above parameters will be explained in detail in “Appendix A”.

**Figure A1:**
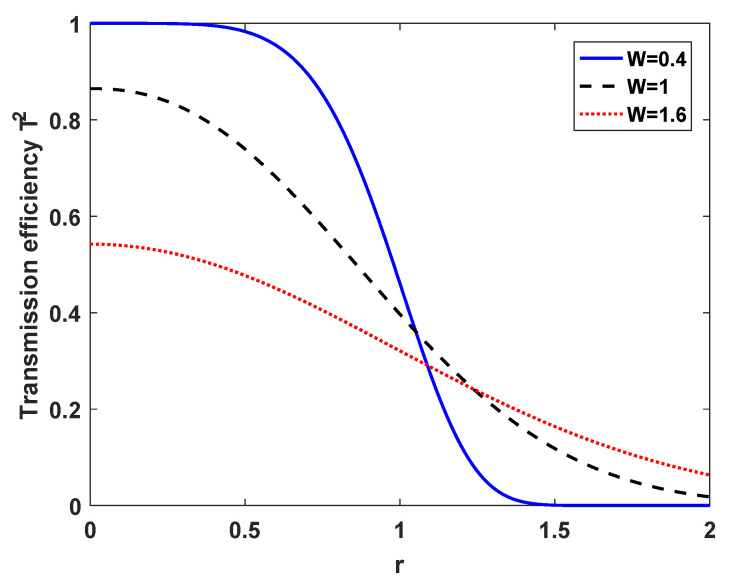
Transmission efficiency $T^{2}$ as a function of the beam deflection distance *r* for different values of the beam-spot radius *W*. The aperture radius *a* is normalized.

Designed for simplicity, the aperture radius *a* is normalized, so that the distribution of $T^{2}$ is acquired. As shown in Figure A1, $T^{2}$ decreases with the increase of beam deflection distance, while the maximum increases with the falling of beam spot radius *W*. On the basic of Ref. [44], the fluctuation of beam-deflection distance *r* is subject to the Rice distribution [45] with the variance $\sigma^{2}$ and the distant *d* from the center of the aperture. Under the circumstance of d=0, this distribution is simplified to the log-negative Weibull distribution,

(3) $$P\left(T\right)=\frac{2R^{2}}{\sigma^{2}\lambda T}{\left(2\ln\frac{T_{0}}{T}\right)}^{2/\lambda -1}\exp\left[-\frac{R^{2}}{2\sigma^{2}}{\left(2\ln\frac{T_{0}}{T}\right)}^{2/\lambda }\right],$$

for $T\in [0,T_{0}]$ and P(T)=0, otherwise. Then, the mean of the fading probability distribution can be calculated from $\langle T\rangle =\int_{0}^{T_{0}}TP\left(T\right)dT$ and $\langle \sqrt{T}\rangle =\int_{0}^{T_{0}}\sqrt{T}P\left(T\right)dT$.

As for strong turbulence, the elliptic-beam model can well describe its characteristics. For now, the probability distribution for free space transmittance could be obtained by using the Glauber–Sudarshan P function on the basic of elliptic beam approximation [24,46]. Compared with the log-normal model [23], the elliptic-beam model is more consistent with the experimental data [47]. Similarly, this model can also be employed to analyze atmospheric quantum channels under different weather circumstances [25].

As depicted in Figure 1b, the elliptic-beam model can describe any spot at the receiving aperture with five parameters, $v={\left(x_{0},y_{0},W_{1},W_{2}\right)}^{T}$ and Φ. Here, $\left(x_{0},y_{0}\right)$ represents the position of the beam centroid and reflects the degree of beam wandering, while $\left(W_{1},W_{2}\right)$ denotes the half axis of elliptical beam section, and Φ is the angle between the half axis $W_{1}$ and the *x*-axis. The above three parameters are used to describe the characteristic of beam broadening and distortion, and give a definition of all possible directions. With this ellipse hypothesis, the transmittance can be approximated as

(4) $$T=T_{0}\exp\left\{-{\left[\frac{r/a}{R(\frac{2}{W_{eff}\left(\Phi -\phi_{0}\right)})}\right]}^{\lambda [2/W_{eff}\left(\Phi -\phi_{0}\right)]}\right\},$$

where *a* denotes the aperture radius, $W_{eff}\left(▪\right)$ represents the effective point radius, $T_{0}$ denotes the transmission coefficient maximum for the centered beam, and R(▪) and λ(▪) are scale and shape functions, respectively. The above parameters will be explained in detail in “Appendix A”.

**Figure 2:**
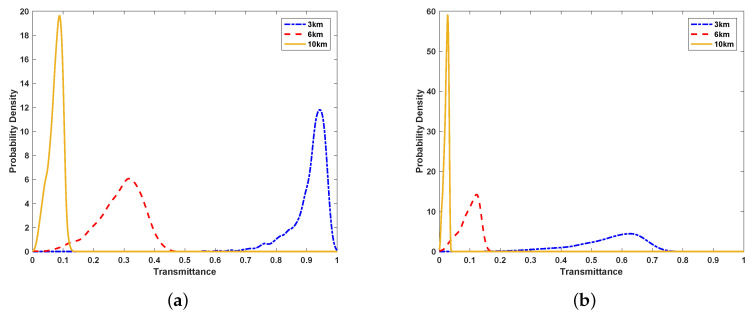
(**a**) the probability density function of transmittance at a distance of 3 km, 6 km, and 10 km for the case of $C_{n}^{2}=2.03\times 10^{-15}$; (**b**) the probability density function of transmittance at a distance of 3 km, 6 km, and 10 km for the case of $C_{n}^{2}=7.46\times 10^{-15}$. The initial beam-spot radius $W_{0}=20$ mm, and the receiving aperture radius a=60 mm.

On the basic of the above equations, the probability distribution of atmospheric transmittance can be calculated by the Monte Carlo method. It is evident that the transmittance *T* is at rest with five parameters $\left\{x_{0},y_{0},\Theta_{1},\Theta_{2},\varphi \right\}$, where $W_{i}^{2}=W_{0}^{2}\exp\Theta_{i}$. Parameter φ is uniformly distributed and irrelevant to the other parameters. The vector $v={\left(x_{0},y_{0},\Theta_{1},\Theta_{2}\right)}^{T}$ is a Gaussian random vector. The above-mentioned parameters are elaborated in “Appendix A”. The density distribution of the horizontal link transmittance is simulated by the Monte Carlo method, which is shown in Figure 2. It is obvious that the distance and refraction index structure parameter $C_{n}^{2}$ affect the transmittance. With the increase of distance and $C_{n}^{2}$, the transmittance declines.

The average value of the PDT could be represented by the simulated transmission value [25]

(5) $$\langle f\left(T\right)\rangle \approx \frac{1}{N}\sum_{i=1}^{N}f\left(\chi_{\mathrm{ext}}T\left(v_{i},\varphi_{i}\right)\right),$$

where $T(v_{i},\varphi_{i})$ is acquired from Equation (4), and the absorption and scattering losses describe with extinction factor $\chi_{\mathrm{ext}}\in \left[0,1\right]$ which is a stochastic variable [25]. Hence, the fading transmittance’s average value has access to acquire

(6) $$\begin{array}{ccc} \langle T\rangle & = & \chi_{\mathrm{ext}}\frac{1}{N}\sum_{i=1}^{N}T\left(v_{i},\varphi_{i}\right), \end{array}$$

(7) $$\begin{array}{ccc} \langle \sqrt{T}\rangle & = & \sqrt{\chi_{\mathrm{ext}}}\frac{1}{N}\sum_{i=1}^{N}\sqrt{T(v_{i},\varphi_{i})}. \end{array}$$

### 2.2. Secret Key Rate in the Atmosphere Turbulence Channel

**Figure 3:**
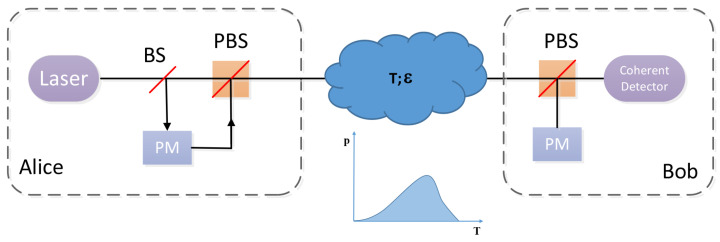
Schematic diagram of prepare-and-measure discrete modulated continuous variable quantum key distribution (CVQKD) in the atmosphere turbulence channel. Alice discretely modulates the quantum signal, and Bob performs the coherent detection on the received states. PM, phase modulator, BS, beamsplitter, and PBS, polarization beamsplitter.

For the sake of the CVQKD investigation in the atmospheric channel, we firstly make analysis centering around the key rate through the fading channel. The schematic diagram of discrete modulated CVQKD in the atmosphere turbulence channel is shown in Figure 3. First, Alice discretely modulates the quantum signal and then dispatches it to Bob via a fading channel whose feature is transmittance distribution *T*. After taking over the quantum signal, Bob conducts the coherent detection on the received signals and acquires the raw key data. A practical detector is featured by an efficiency η and a noise $v_{el}$ on account of detector electronics.

Here, the reachable secret key rate of atmospheric discrete modulation coherent state CVQKD is presented on the basis of the calculation results of the above sections. In the case of collective attack, assume that Alice and Bob apply the reverse reconciliation with reconciliation efficiency β; the key rate can be given by [48]

(8) K=βI(a:b)−χ(b:E).

With the assumption of channel’s transmittance $\langle T\rangle ,\langle \sqrt{T}\rangle$ and excess noise ε, the covariance matrix of $\rho_{AB}$ is shown as [49]

(9) $$\gamma_{AB}=\left(\begin{array}{cc} (V_{A}+1)I & \langle \sqrt{T}\rangle Z\sigma_{z}\\ \langle \sqrt{T}\rangle Z\sigma_{z} & [\langle T\rangle \left(V_{A}+\varepsilon \right)+1]I \end{array}\right),$$

where $Z=V_{A}\left(\xi_{0}^{3/2}\xi_{1}^{-1/2}+\xi_{1}^{3/2}\xi_{2}^{-1/2}+\xi_{2}^{3/2}\xi_{3}^{-1/2}+\xi_{3}^{3/2}\xi_{0}^{-1/2}\right)$, with

(10) $$\begin{array}{c} \xi_{0,2}=\frac{1}{2}\exp\left(-0.5V_{A}\right)\left[\cosh\left(-0.5V_{A}\right)\pm \cos\left(-0.5V_{A}\right)\right],\\ \xi_{1,3}=\frac{1}{2}\exp\left(-0.5V_{A}\right)\left[\sinh\left(-0.5V_{A}\right)\pm \sin\left(-0.5V_{A}\right)\right]. \end{array}$$

**Figure 4:**
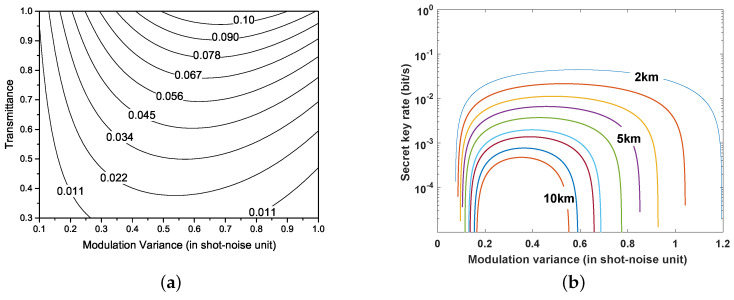
(**a**) key rate as a function of modulation variance $V_{A}$ and transmittance; (**b**) key rate as a function of modulation variance $V_{A}$ for various distances. From top to bottom, the distance increases by 1 km. The excess noise ε is set as 0.01, detector efficiency η=0.6, electrical noise $v_{el}=0.01$.

According to covariance matrix, $\gamma_{AB}$, I(a:b) and χ(b:E) can be acquired (see Appendix B). Figure 4 shows the secret key rate versus the transmittance and modulation variance. The results indicate that the maximum key rate can be obtained by adjusting the modulation variance. Therefore, the optimal modulation variance $V_{A}$ can be derived by maximizing the key rate under diverse system conditions. The system conditions include the transmission distance *L*, the Rytov variance $\sigma_{R}^{2}$, the detector efficiency η, and the electrical noise $v_{el}$.

In this section, we have analyzed the transmittance of the atmospheric channel under different turbulence intensity and calculate the secret key rate of discrete modulated CVQKD in the atmospheric channel. Through the above analysis, we conclude that adjusting the modulation variance can increase the secret key rate of the system.

## 3. BPNN-Based CVQKD Scheme

Different from optical fiber channel, the transmittance of the atmospheric channel is nonlinear. Factors affecting transmittance include not only distance but also climatic conditions. In order to achieve the maximum secret key rate transmission, it is very important to choose an appropriate modulation variance at Alice end. BPNN can be applied to the system to predict the most suitable modulation variance $V_{A}$ quickly and accurately. For simplicity, we convert the above relationship into the mathematical formula as follows:

(11) $$\begin{array}{c} \vec{u}=\left(L,\sigma_{R}^{2},\langle T\rangle \right).\\ \vec{v}=\left(V_{A}\right). \end{array}$$

The secret key rate *K* is the function of $V_{A}$, which is $K=f(\vec{v})$. Modulation variance optimization could be regarded as searching for $\vec{v_{opt}}$ to maximize *K*, as indicated

(12) $$\vec{v_{opt}}=argmax_{\vec{v}\in V}\left[K=f\left(\vec{v}\right)\right].$$

At the beginning, we invent an input layer that contains three neurons. They receive distance *L*, Rytov parameter $\sigma_{R}^{2}$ and transmittance 〈T〉 as the input, respectively. For input parameters, *L* is the distance between Alice and Bob, and Rytov parameter $\sigma_{R}^{2}$ is mainly dependent on refraction index structure parameter $C_{n}^{2}$. Transmittance 〈T〉 is relevant to atmosphere turbulence. Then, a hidden layer comprised of eight neurons is enhanced to connect the input layer. In the end, an output layer of only one neuron is installed, which will output the modulation variance $V_{A}$.

Here, we prepare the training data for BPNN. First, a program was written to randomly sample the input data space, randomly select the combination of $\vec{u}$ (for which we generate 1000 sets of data from L=0−11km, $\sigma_{R}^{2}=0-20$, 〈T〉=0−1), and calculate the corresponding optimal parameters $\vec{v}$ and the secret key rate by using the local search algorithm (LSA).

The next step is to train BPNN. We record all the above-mentioned $\vec{u}$ and $\vec{v}$ as the training dataset *U* and *V*. Then, we introduce the BPNN that is applied to predict $V_{A}$. First, we extract $V_{A}$ from $\vec{v_{opt}}$ and make a label dataset $V_{1}$. Then, we put the dataset *U* into BPNN and adjust the network connection weights with the back propagation algorithm (BPA) according to the difference between the output of the BPNN and label dataset $V_{1}$. The working principle of BPNN is described in detail in Appendix C. Once trained, the neural network can find the optimal parameters and key rate directly according to any input, which greatly speeds up the parameter optimization process.

**Figure 5:**
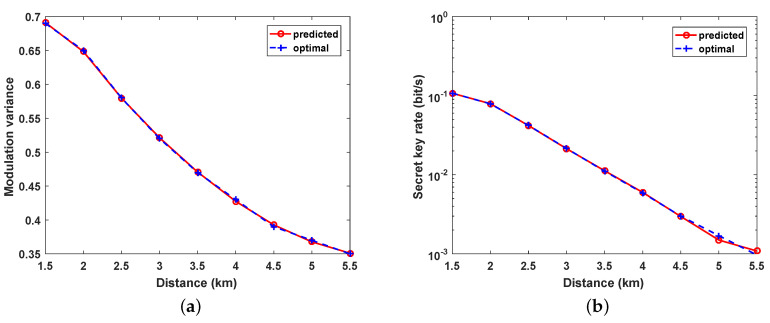
(**a**) comparison of BPNN-predicted and optimized modulation variance $V_{A}$; (**b**) comparison of the key rate generated using the BPNN predicted parameter and optimized key rate. The excess noise ε is set as 0.01, detector efficiency η=0.6, and electrical noise $v_{el}=0.01$.

After the training is achieved, three groups of data are randomly selected using the training network, and the outcomes are recorded in Table 2. The table indicates that the predicted parameters and the matching key rate are very familiar with the optimal values acquired by LSA, for the parameters predicted by BPNN reaching 99.99% of the optimal key rate. As depicted in Figure 5, we compared the BPNN-predicted and optimized parameters by scanning the distance from 0 to 11 km. Compared with the traditional LSA, the BPNN performs very well in predicting optimal values for modulation variance and attains a very similar key rate level.

## 4. Performance Analysis

In this part, we analyze the key rate that can be realized according to the consequences of Section 2 and Section 3. The secret key rate is evaluated by the Monte Carlo method. The excess noise induced by the phase fluctuation is incapable of compensating precisely yet, and after adopting diverse valid compensation approaches, it is tough to evaluate the actual excess noise [50]. Therefore, we ignore the change of phase excess noise free space situations temporarily. However, we still study the key rates that can be achieved at different fixed excess noise levels: ε=0.01 and ε=0.03.

**Figure 6:**
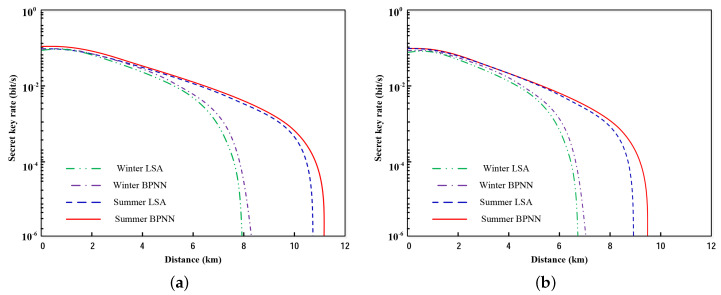
The secret key rate as a function of distance for local search algorithm (LSA) and BPNN in the summer and winter. (**a**) ε=0.01; (**b**) ε=0.03.

The secret key rate with excess noise ε=0.01 is illustrated in Figure 6a. As Figure 6a shows, the secret key rate of the system employing BPNN is higher than LSA, a little higher but not much. Then, the excess noise is set as ε=0.03, and the performance is as portrayed in Figure 6b. In comparison with Figure 6a, the realized transmission distance is significantly shortened.

The performance profiling in this section points out a few of pivotal cores. Above all, the BPNN based CVQKD system furnishes a higher achievable key rate and more efficient parameter optimization. Secondly, we find that transmittance fluctuations are negative to key rates. Therefore, the effect of beam wandering, broadening and deformation should be emphasized in practical experiments. Third, due to the considerable influence of excess noise, the phase excess noise will be much larger than 0.03; valid approaches to possess the phase excess noise are required to improve the key rate.

## 5. Conclusions

In this work, considering that the fluctuating of the atmospheric turbulence channel has significant influences on the performance and practical security of CVQKD system, we put forward a method to optimize the relevant system. Here, we employ machine learning to CVQKD, which could adjust the modulation variance to an optimal value to ensure the system security and make the system performance optimal. The transmittance is simulated in consideration of not only the atmospheric turbulence, but also the absorption and scattering losses. The LSA algorithm is adopted to generate optimal data as training data of BPNN. Meanwhile, by comparing the two algorithms, we get that a BPNN based CVQKD system furnishes a higher achievable key rate and more efficient parameter optimization. In particular, this approach can be applied to any measurable physical parameter of signals in atmospheric turbulence or fiber channel [35,39].

## Figures and Tables

**Table 1:** The values (median) of $C_{n}^{2}$ in four seasons.

	Spring	Summer	Autumn	Winter
$C_{n}^{2}\;\left(m^{-2/3}\times 10^{-15}\right)$	2.03	2.12	5.56	7.46

**Table 2:** Optimal vs. back-propagation neural network (BPNN)-predicted parameters. The modulation variance $V_{A}$ is the BPNN-predicted parameter, and key rate *K* is generated by applying the BPNN predicted $V_{A}$. The parameters *L*, $\sigma_{R}^{2}$, 〈T〉 and $C_{n}^{2}$ are the systems conditions. Moreover, $C_{n}^{2}$ and $\sigma_{R}^{2}$ can be converted by the formula $\sigma_{R}^{2}=1.23C_{n}^{2}k^{\left(7/6\right)}L^{\left(11/9\right)}$, hence the input dimension is 3 instead of 4.

Method	L	$\sigma_{R}^{2}$	〈T〉	$C_{n}^{2}$	$V_{A}$	K
Optimized	2 km	2.3269	0.8597	$2\times 10^{-15}$	0.70	0.2550
BPNN	2 km	2.3269	0.8597	$2\times 10^{-15}$	0.6975	0.2556
Optimized	4 km	8.2922	0.3859	$2\times 10^{-15}$	0.1160	0.0388
BPNN	4 km	8.2922	0.3859	$2\times 10^{-15}$	0.1161	0.0391

**Figure A2:**
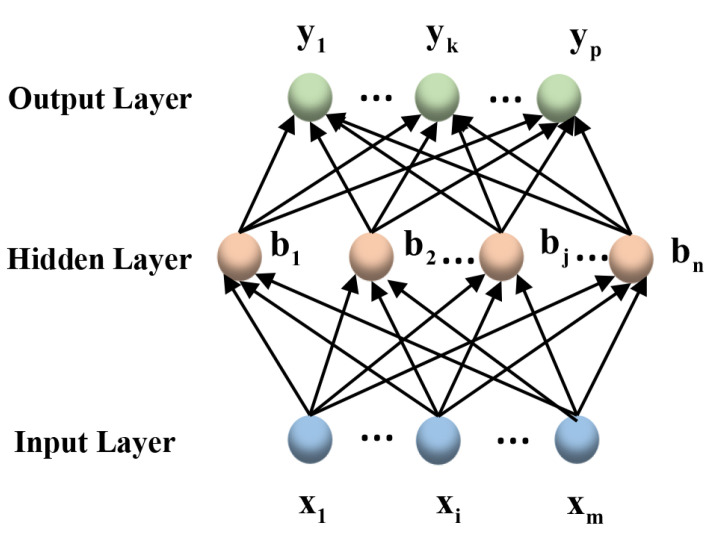
The basic structure of BPNN. It contains three layers, namely the input layer, the hidden layer and the output layer.

**Figure A3:**
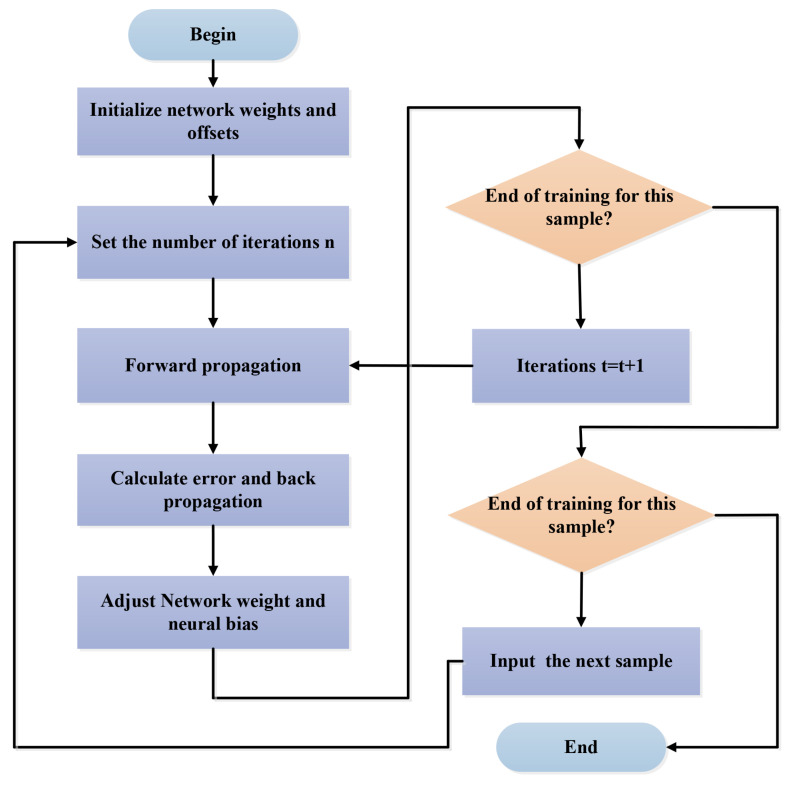
The flow diagram of the back-propagation neural network algorithm.

## Appendix A. The Atmospheric Transmittance Analysis

In this appendix, we will demonstrate the formulas for the aforementioned parameters in Section 2.1. Under the circumstance of beam wandering (see Figure 1a), an approximate analytical representative of transmission efficiency can be expressed as

(A1) $$T^{2}=T_{0}^{2}\exp\left[-{\left(\frac{r}{R}\right)}^{\lambda }\right],$$

where *r* denotes the distance of beam-deflection, λ and *R* are the shape and scale parameter, respectively, which can be derived

(A2) $$\begin{array}{cc} \lambda = & 8\frac{a^{2}}{W^{2}}\frac{\exp\left[-4\frac{a^{2}}{W^{2}}\right]I_{1}\left(4\frac{a^{2}}{W^{2}}\right)}{1-\exp\left[-4\frac{a^{2}}{W^{2}}\right]I_{0}\left(4\frac{a^{2}}{W^{2}}\right)}\\ \; & \times {\left[\ln\left(\frac{2T_{0}^{2}}{1-\exp\left[-4\frac{a^{2}}{W^{2}}\right]I_{0}\left(4\frac{a^{2}}{W^{2}}\right)}\right)\right]}^{-1}, \end{array}$$

(A3) $$R=a{\left[\ln\left(\frac{2T_{0}^{2}}{1-\exp\left[-4\frac{a^{2}}{W^{2}}\right]I_{0}\left(4\frac{a^{2}}{W^{2}}\right)}\right)\right]}^{-(1/\lambda )},$$

and $T_{0}$ denotes the transmission coefficient maximum for the specified beam-spot radius value *W*; one can be given by

(A4) $$T_{0}^{2}=1-\exp\left[-2\frac{a^{2}}{W^{2}}\right],$$

where *a* denotes the aperture radius. Obviously, the transmittance efficiency $T^{2}$ is dominated by the ratio a/W and reduced at $T_{0}$. Designed for simplicity, the aperture radius *a* is normalized, so that the distribution of $T^{2}$ is acquired.

For strong turbulence, the maximal transmittance for a centered beam $T_{0}$ can be given by

(A5) $$\begin{array}{cc} T_{0}= & 1-I_{0}\left(a^{2}\frac{W_{1}^{2}-W_{2}^{2}}{W_{1}^{2}W_{2}^{2}}\right)\exp\left(-a^{2}\frac{W_{1}^{2}+W_{2}^{2}}{W_{1}^{2}W_{2}^{2}}\right)\\ \; & -2\left\{1-\exp\left[-\frac{a^{2}}{2}\left(\frac{1}{W_{1}}-\frac{1}{W_{2}}\right)\right]\right\}\\ \; & \times \exp\left[-{\left\{\frac{\frac{{\left(W_{1}+W_{2}\right)}^{2}}{|W_{1}^{2}-W_{2}^{2}|}}{R(\frac{1}{W_{1}}-\frac{1}{W_{2}})}\right\}}^{\lambda (1/W_{1}-1/W_{2})}\right], \end{array}$$

which is a function of the two eigenvalues $W_{i}^{2},i=1,2$. The shape λ and scale *R* functions are given by

(A6) $$\begin{array}{cc} \lambda (\xi )= & 2a^{2}\xi^{2}\frac{\exp\left[-a^{2}\xi^{2}\right]I_{1}\left(a^{2}\xi^{2}\right)}{1-\exp\left[-a^{2}\xi^{2}\right]I_{0}\left(a^{2}\xi^{2}\right)}\\ \; & \times {\left[\ln(2\frac{1-\exp[-0.5a^{2}\xi^{2}]}{1-\exp\left[-a^{2}\xi^{2}\right]I_{0}\left(a^{2}\xi^{2}\right)})\right]}^{-1}, \end{array}$$

(A7) $$R\left(\xi \right)={\left[\ln\left(2\frac{1-\exp[-0.5a^{2}\xi^{2}]}{1-\exp\left[-a^{2}\xi^{2}\right]I_{0}\left(a^{2}\xi^{2}\right)}\right)\right]}^{-(1/\lambda (\xi ))}.$$

In the case of $\varphi =\Phi -\phi_{0}$, the effective squared spot radius is expressed as

(A8) $$\begin{array}{cc} W_{eff}^{2}\left(\varphi \right)= & 4a^{2}\{W(\frac{4a^{2}}{W_{1}W_{2}}\exp\left[\frac{a^{2}}{W_{1}^{2}}\left(1+2\cos^{2}\left(\varphi \right)\right)\right]\\ \; & \times \exp\left[\frac{a^{2}}{W_{2}^{2}}\left(1+2\sin^{2}\left(\varphi \right)\right)\right])\}. \end{array}$$

On the basic of the above equations, the probability distribution of atmospheric transmittance can be calculated by the Monte Carlo method. It is evident that the transmittance *T* is decided by five parameters $\left\{x_{0},y_{0},\Theta_{1},\Theta_{2},\varphi \right\}$, where $W_{i}$ is log-normally distributed and it can be acquired by $W_{i}^{2}=W_{0}^{2}\exp\Theta_{i}$. Parameter φ is uniformly distributed and irrelevant to the other parameters. The relevance of $\left\{x_{0},y_{0},\Theta_{1},\Theta_{2}\right\}$ is represented by covariance matrix [24,25]

(A9) $$\Xi =\left(\begin{array}{cccc} \langle x_{0}^{2}\rangle & 0 & 0 & 0\\ 0 & \langle x_{0}^{2}\rangle & 0 & 0\\ 0 & 0 & \langle \Theta_{1}^{2}\rangle & \langle \Theta_{1}\Theta_{2}\rangle \\ 0 & 0 & \langle \Theta_{1}\Theta_{2}\rangle & \langle \Theta_{2}^{2}\rangle \end{array}\right).$$

For weak turbulence, the elements of the covariance matrix Ξ can be given by [24]

(A10) $$\begin{array}{c} \begin{array}{cc} \langle x_{0}^{2}\rangle =\langle y_{0}^{2}\rangle & =0.33W_{0}^{2}\sigma_{R}^{2}\Omega^{-\frac{7}{6}},\\ \langle \Theta_{1}^{2}\rangle =\langle \Theta_{2}^{2}\rangle & =\ln\left[1+\frac{1.2\sigma_{R}^{2}\Omega^{\frac{5}{6}}}{{\left(1+2.96\sigma_{R}^{2}\Omega^{\frac{5}{6}}\right)}^{2}}\right],\\ \langle \Theta_{1}\Theta_{2}\rangle & =\ln\left[1-\frac{0.8\sigma_{R}^{2}\Omega^{\frac{5}{6}}}{{\left(1+2.96\sigma_{R}^{2}\Omega^{\frac{5}{6}}\right)}^{2}}\right], \end{array} \end{array}$$

with

(A11) $$\begin{array}{c} \begin{array}{cc} \Omega & =\frac{kW_{0}^{2}}{2L},\\ \langle \Theta_{1,2}\rangle & =\ln\left[\frac{{\left(1+2.96\sigma_{R}^{2}\Omega^{\frac{5}{6}}\right)}^{2}}{\Omega^{2}\sqrt{{\left(1+2.96\sigma_{R}^{2}\Omega^{\frac{5}{6}}\right)}^{2}+1.2\sigma_{R}^{2}\Omega^{\frac{5}{6}}}}\right]. \end{array} \end{array}$$

In the case of strong turbulence, these elements are expressed by [24]

(A12) $$\begin{array}{c} \begin{array}{cc} \langle x_{0}^{2}\rangle =\langle y_{0}^{2}\rangle & =0.75W_{0}^{2}\sigma_{R}^{\frac{8}{5}}\Omega^{-1},\\ \langle \Theta_{1}^{2}\rangle =\langle \Theta_{2}^{2}\rangle & =\ln\left[1+\frac{13.14\gamma \sigma_{R}^{\frac{12}{5}}\Omega^{-1}}{{\left(\gamma +1.71\sigma_{R}^{\frac{12}{5}}\Omega^{-1}-2.99\sigma_{R}^{\frac{8}{5}}\Omega^{-1}\right)}^{2}}\right],\\ \langle \Theta_{1}\Theta_{2}\rangle & =\ln\left[1+\frac{0.65\gamma \sigma_{R}^{\frac{12}{5}}\Omega^{-1}}{{\left(\gamma +1.71\sigma_{R}^{\frac{12}{5}}\Omega^{-1}-2.99\sigma_{R}^{\frac{8}{5}}\Omega^{-1}\right)}^{2}}\right], \end{array} \end{array}$$

with

(A13) $$\begin{array}{c} \begin{array}{cc} \gamma & =\frac{1+\Omega^{2}}{\Omega^{2}},\\ \langle \Theta_{1,2}\rangle & =\ln\left[\frac{{\left(\gamma +1.71\sigma_{R}^{\frac{12}{5}}\Omega^{-1}-2.99\sigma_{R}^{\frac{8}{5}}\Omega^{-1}\right)}^{2}}{\sqrt{{\left(\gamma \;+\;1.71\sigma_{R}^{\frac{12}{5}}\Omega^{-1}\;-\;2.99\sigma_{R}^{\frac{8}{5}}\Omega^{-1}\right)}^{2}\;+3.24\gamma \sigma_{R}^{\frac{8}{5}}\Omega^{-1}}}\right]. \end{array} \end{array}$$

The weak turbulence results can be applied, e.g., for short propagation distances with $\sigma_{R}^{2}<1$. In near-to-ground propagation, the latter condition is fulfilled for optical frequencies for night-time communication. The strong turbulence results are applied for short distance communication, $\sigma_{R}^{2}\gg 1$. For a near-to-ground communication scenario, this corresponds to the day-time operation on clear sunny days.

## Appendix B. Secret Key Rate

With the assumption of channel’s transmittance $\langle T\rangle ,\langle \sqrt{T}\rangle$ and excess noise ε, the covariance matrix of $\rho_{AB}$ is shown as [49]

(A14) $$\gamma_{AB}=\left(\begin{array}{cc} (V_{A}+1)I & \langle \sqrt{T}\rangle Z\sigma_{z}\\ \langle \sqrt{T}\rangle Z\sigma_{z} & [\langle T\rangle \left(V_{A}+\varepsilon \right)+1]I \end{array}\right).$$

According to $\gamma_{AB}$, the mutual information of Alice and Bob for homodyne detection is

(A15) $$I\left(a:b\right)=\frac{1}{2}\log_{2}\frac{1}{1-\frac{{\langle \sqrt{T}\rangle }^{2}V_{A}}{\langle T\rangle \left(V+\chi_{tot}\right)}},$$

where $\chi_{tot}=\frac{\left(1+\upsilon_{el}\right)}{\eta \langle T\rangle }-1+\varepsilon$.

The maximum information that Eve can be accessed χ(b:E) is expressed by

(A16) $$\chi \left(b:E\right)=\sum_{i=1}^{2}G\left(\frac{\lambda_{i}-1}{2}\right)-\sum_{i=3}^{5}G\left(\frac{\lambda_{i}-1}{2}\right),$$

where $G\left(x\right)=\left(x+1\right)\log_{2}\left(x+1\right)-x\log_{2}x$, and the formulas to calculate $\lambda_{i},i=1,2,\ldots ,5$ are derived from

(A17) $$\begin{array}{ccc} \lambda_{1,2}^{2} & = & \frac{1}{2}\left(A\pm \sqrt{A^{2}-4B}\right), \end{array}$$

(A18) $$\begin{array}{ccc} \lambda_{3,4}^{2} & = & \frac{1}{2}\left(C\pm \sqrt{C^{2}-4D}\right), \end{array}$$

(A19) $$\begin{array}{ccc} \lambda_{5} & = & 1, \end{array}$$

with

(A20) $$\begin{array}{cc} A & =V^{2}+{\langle T\rangle }^{2}{\left(V+\chi_{line}\right)}^{2}-2\langle T\rangle Z^{2},\\ B & ={\left(\langle T\rangle V^{2}+\langle T\rangle V\chi_{line}-{\langle \sqrt{T}\rangle }^{2}Z^{2}\right)}^{2}, \end{array}$$

(A21) $$\begin{array}{cc} C & =\frac{AX_{h}+V\sqrt{B}+\sqrt{B}}{\langle T\rangle \left(V+\chi_{tot}\right)},\\ D & =\sqrt{B}\frac{V+\sqrt{B}\chi_{h}}{\langle T\rangle \left(V+\chi_{tot}\right)}, \end{array}$$

where $\chi_{line}=1/\langle T\rangle -1+\varepsilon$, $\chi_{h}=\left(1-\eta +\upsilon_{el}\right)/\eta .$

## Appendix C. Back-Propagation Neural Network

The back-propagation neural network (BPNN) is the most elementary and widely used neural network. Its output result adopts forward propagation while the error takes in back propagation. The feedforward neural network refers to the hierarchical arrangement of neurons, which is made up of input layer, hidden layer and output layer. Neurons in each layer of this neural network only receive input from neurons in the previous layer, and the latter layer has no signal feedback to the former layer. Each layer converts the input data to some extent, and then utilizes the outcome as the input of the following layer up to the eventual outcome. The back propagation is used to adjust the network weights and thresholds during training, which needs to be supervised. When your network is not well trained, the output must be different from what you think; then, we will get a deviation, and the deviation of one level forward, layer by layer to get the error $\delta^{\left(i\right)}$. This is the feedback. The feedback is used to find the partial derivative, which is typically used for gradient descent. Then, the gradient descent is applied to acquire the minimum value of the cost function, so that the error between the expectation and the output can be reduced as much as possible.

**Table A1:** Parameter definition.

Parameter	Definition
$\theta_{k}$	Threshold of the *k*-th neuron in output layer
$\gamma_{j}$	Threshold of the *j*-th neuron in hidder layer
$v_{ij}$	The weight between the *i*-th node in the input layer and the *j*-th node in the hidden layer
$w_{jk}$	The weight between the *j*-th node in the hidden layer and the *k*-th node in the output layer
$\alpha_{j}$	The input value that the *j*-th neuron received in the hidden layer
$\beta_{k}$	The input value that the *k*-th neuron received in the output layer
f(x)	Activation function.
η	Learning rate

The flow diagram of the back-propagation neural network algorithm is shown in Figure A3, it can be roughly divided into five steps:

**Step 1: Initialize network weights and offsets.** We know that, after training, the connection weight (network weight) between different neurons is diverse. Therefore, in the initialization stage, we give each network connection weight a small random number (generally −1.0~1.0 or −0.5~0.5), and each neuron has a bias (bias can be regarded as the weight of each neuron), which will also be initialized to a random number.

**Step 2: Forward propagation.** Enter a training pattern and figure out the output result of each and every neuron. Each neuron works out a linear combination of its inputs. The calculation formula of the activation value of each neuron in the hidden layer is indicated as

(A22) $$\alpha_{j}=\sum_{i=1}^{m}v_{ij}x_{i}.$$

Then, the output value of the *j*-th neuron in the hidden layer is $f(\alpha_{j}-\gamma_{j})$, where $f\left(x\right)=\frac{1}{1+e^{-x}}$. The input value that the *k*-th neuron received in the output layer is

(A23) $$\beta_{k}=\sum_{j=1}^{n}w_{jk}b_{j}.$$

In the end, the final output of the *k*-th neuron in the output layer is

(A24) $$y_{k}=f\left(\beta_{k}-\theta_{k}\right).$$

**Step 3: Calculation error and back propagation.** This stage is the learning process of the algorithm. In the learning process, we hope that the output of the algorithm can be consistent with our real value to the greatest extent. When the output value and real value is different, there will ineluctably be an error. The lesser the error, the better the prediction effect of the algorithm. Obviously, the input data are known, and the variables are only those connection weights that will affect the output.

For training example $\left(x_{h},y_{h}\right)$, suppose the output of the neural network is ${\hat{y}}_{h}=\left({\hat{y}}_{1}^{h},{\hat{y}}_{2}^{h},\ldots ,{\hat{y}}_{p}^{h}\right)$. Therefore, the mean square error in the training example $\left(x_{h},y_{h}\right)$ is expressed as

(A25) $$E_{h}=\frac{1}{2}\sum_{k=1}^{p}{\left({\hat{y}}_{k}^{h}-y_{k}^{h}\right)}^{2}.$$

Based on the gradient descent strategy, the BP algorithm adjusts the parameters in the direction of the negative gradient of the target. In the case of learning rate η, for the error $E_{h}$ in the formula Equation (A25), there is the following formula:

(A26) $$\begin{array}{cc} \Delta w_{jk} & =-\eta \frac{\partial E_{h}}{\partial w_{jk}}\\ \; & =\eta g_{k}bj, \end{array}$$

where

(A27) $$g_{k}={\hat{y}}_{k}^{h}\left(1-{\hat{y}}_{k}^{h}\right)\left(y_{k}^{h}-{\hat{y}}_{k}^{h}\right).$$

Similarly, $\Delta \theta_{k}$, $\Delta v_{ij}$ and $\Delta \gamma_{j}$ can be derived

(A28) $$\begin{array}{cc} \Delta \theta_{k} & =-\eta g_{k}, \end{array}$$

(A29) $$\begin{array}{cc} \Delta v_{ij} & =\eta e_{j}x_{i}, \end{array}$$

(A30) $$\begin{array}{cc} \Delta \gamma_{j} & =-\eta e_{j}, \end{array}$$

with

(A31) $$\begin{array}{cc} e_{j} & =-\frac{\partial E_{h}}{\partial \alpha_{j}}\\ \; & =bj\left(1-b_{j}\right)\sum_{k=1}^{p}w_{jk}g_{k}. \end{array}$$

**Step 4: Adjust connection weights and thresholds.** At this stage, the connection weight and threshold are adjusted in accordance with the error of hidden layer neurons.

**Step 5: End of the training.** For each sample, if its error is less than the set threshold or the number of iterations has been reached, then the training is over. Otherwise, the training is resumed at the second step.
